# Atypical Adenomatous Hyperplasia as a Precursor of Lung Adenocarcinoma

**DOI:** 10.7759/cureus.89394

**Published:** 2025-08-05

**Authors:** Gonçalo T Lopes, Bernardo Bem, Diogo Mendonça, Marcos Oliveira, Gonçalo Samouco, Madalena Reis, Joana Ribeiro, Luís Ferreira

**Affiliations:** 1 Pulmonology, Unidade Local de Saúde (ULS) da Guarda, Guarda, PRT

**Keywords:** adenocarcinoma, atypical adenomatous hyperplasia, biopsy, cancer, precursor

## Abstract

Pulmonary atypical adenomatous hyperplasia (AAH) is a recognized precursor lesion to pulmonary adenocarcinoma (ADC). We present the case of a 79-year-old ex-smoker in whom transthoracic needle biopsy revealed histological features suggestive of lung ADC. However, surgical resection of the lesion later demonstrated only AAH. This discrepancy likely reflects sampling variability or partial lesion regression, emphasizing the diagnostic challenges posed by small pulmonary nodules. The case highlights the importance of integrating histopathological, radiological, and clinical data, and underscores the critical role of multidisciplinary decision-making in managing preinvasive pulmonary lesions.

## Introduction

Lung cancer remains one of the leading causes of cancer-related mortality worldwide, with adenocarcinoma (ADC) being the most common histological subtype. Among its recognized precursor lesions, pulmonary atypical adenomatous hyperplasia (AAH) has gained increasing clinical relevance due to its potential for malignant transformation. AAH is defined as a small (≤5 mm) lesion composed of mildly atypical cuboidal to columnar epithelial cells lining the alveolar septa, resembling type II pneumocytes or Clara (club) cells [1,2].

AAH is typically detected incidentally on imaging, often presenting as a focal ground-glass opacity (GGO) on high-resolution computed tomography (CT). Hospital-based studies estimate its presence in up to 20% of surgically resected ADCs [3], though its true prevalence in the general population remains uncertain. With the growing use of low-dose CT screening, increasing numbers of small, indeterminate pulmonary nodules are being identified, some of which may correspond to AAH or early-stage malignancy.

At the molecular level, AAH is believed to represent the earliest stage in a sequential progression toward invasive ADC, evolving through intermediate stages such as ADC in situ and minimally invasive ADC. This progression is thought to involve early genetic alterations and increasing molecular complexity as lesions advance. However, distinguishing indolent from progressive lesions remains a clinical challenge.

Accurate differentiation between AAH and invasive ADC carries important therapeutic implications. While AAH may often be managed conservatively with imaging surveillance, confirmed malignancy typically warrants surgical resection and closer oncologic monitoring. Given the histological subtlety of AAH and potential for sampling limitations, diagnosis requires careful integration of radiologic, histopathologic, and clinical data, best approached through multidisciplinary collaboration.

## Case presentation

A 79-year-old man with a 20-pack-year smoking history presented with unintentional weight loss of approximately 20 kg over six months.

**Figure 1:**
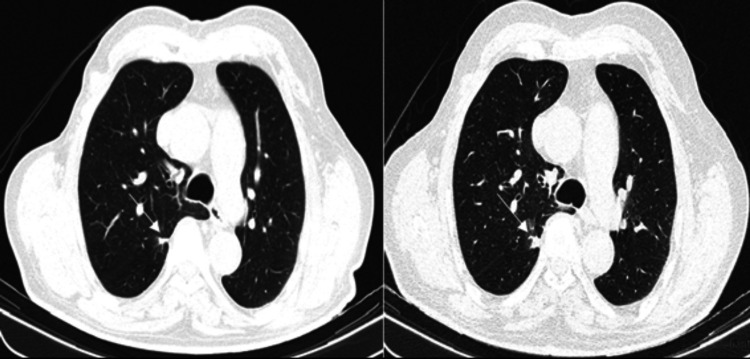
Nodule growth observed on CT scan of the thorax Left: Axial CT scan of the thorax (slice thickness: 3.00 mm, lung window) showing a solitary solid pulmonary nodule (white arrow) measuring 6 mm in the apical segment of the right lower lobe, with spiculated margins and pleural contact. Right: Corresponding axial CT scan one year later demonstrating interval growth of the same nodule (white arrow), now measuring 17 mm.

An initial chest CT scan revealed a solitary, solid pulmonary nodule measuring 6 mm in the apical segment of the right lower lobe. The lesion had spiculated margins and pleural contact, radiological features highly suggestive of malignancy. However, the differential diagnosis for a solitary pulmonary nodule with such characteristics includes both benign and malignant conditions. These range from granulomatous infections, organizing pneumonia, and pulmonary infarction to malignant entities such as primary lung carcinoma or pulmonary metastases. Although the lesion remained stable at 6-month follow-up, a repeat CT at 18 months showed an increase in size to 17 mm, warranting further investigation (Figure 1).

A CT-guided transthoracic needle biopsy was performed despite the lesion’s small size and apical subpleural location, which posed a technical challenge. The decision was justified by the nodule’s solid consistency, peripheral accessibility, and high pre-test probability of malignancy based on radiologic features. The procedure was uneventful, with no complications such as pneumothorax or bleeding.

**Figure 2:**
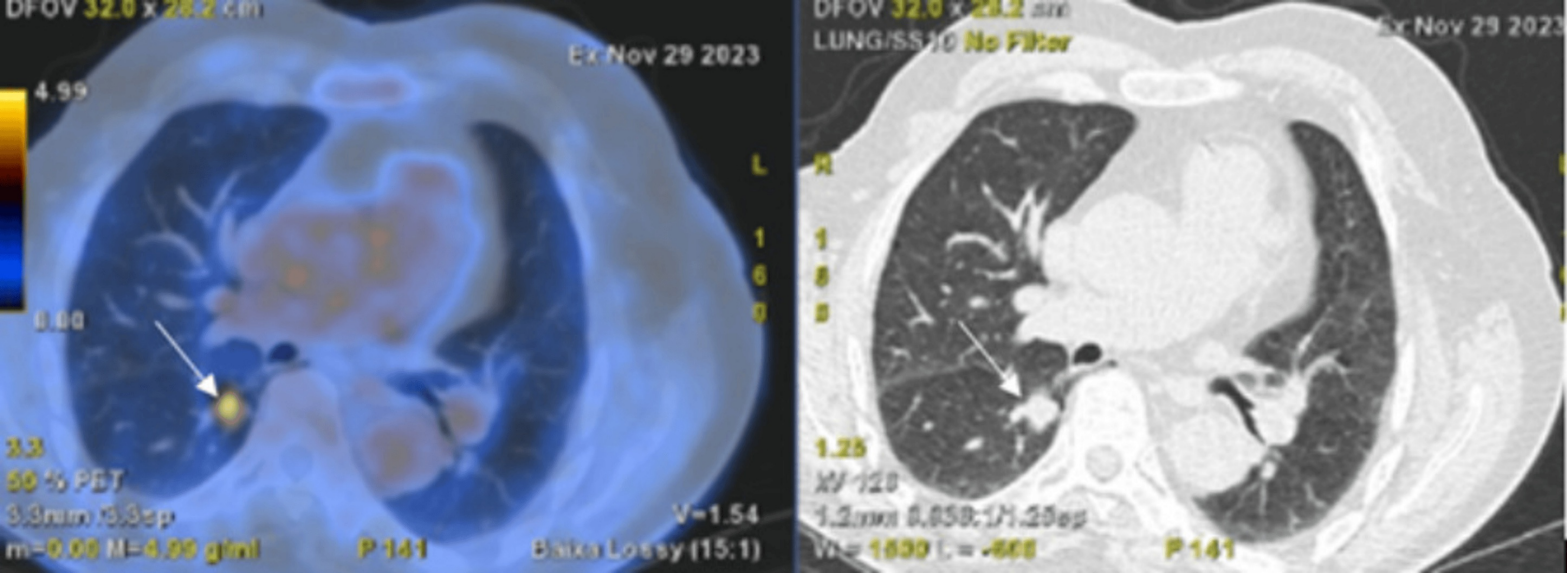
PET/CT scan of the thorax 18F-fluorodeoxyglucose positron emission tomography/computed tomography (18F-FDG PET/CT) scan of the thorax showing increased metabolic activity within the pulmonary nodule (white arrow), highly suggestive of malignancy.

Histopathological evaluation revealed features consistent with pulmonary ADC, exhibiting a lepidic growth pattern with mucinous characteristics and an enteric phenotype. Immunohistochemical staining demonstrated positivity for CK7 and negativity for TTF1, CD56, and CK5/6. PD-L1 expression was low (<1%), and next-generation sequencing identified a KRAS G12A mutation, a known driver mutation in lung ADC. No alterations were detected in EGFR, ALK, ROS1, BRAF, HER2, MET, NTRK1/2/3, or RET. A whole-body 18F-fluorodeoxyglucose positron emission tomography/computed tomography (18F-FDG PET/CT) scan demonstrated increased metabolic activity in the nodule, with a maximum standardized uptake value (SUVmax) of 4.4, supporting the suspicion of malignancy. No evidence of distant metastasis was found, and a primary enteric tumor was ruled out (Figure 2).

**Figure 3:**
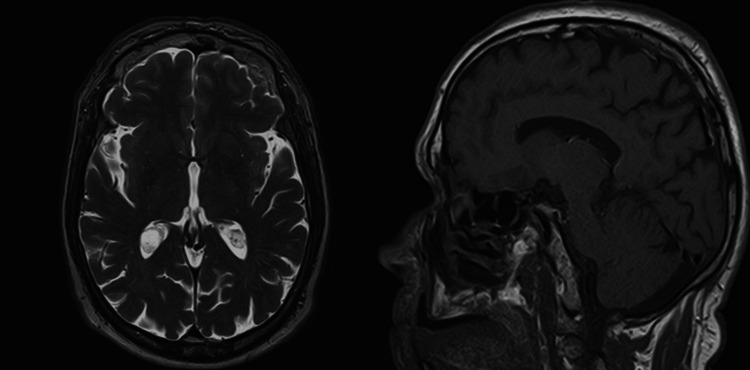
Brain MRI showing no metastasis

Cranial magnetic resonance imaging (MRI) ruled out secondary brain lesions (Figure 3). Based on clinical and imaging findings, the ADC was staged as cT1bN0M0 (stage IA2). Following multidisciplinary discussion and assessment of the patient's surgical fitness, curative resection was recommended. The patient underwent a right lower lobectomy with mediastinal lymphadenectomy via video-assisted thoracoscopic surgery. However, histopathological examination of the resected specimen revealed only a 3.5 mm whitish lesion consistent with AAH. 

**Figure 4:**
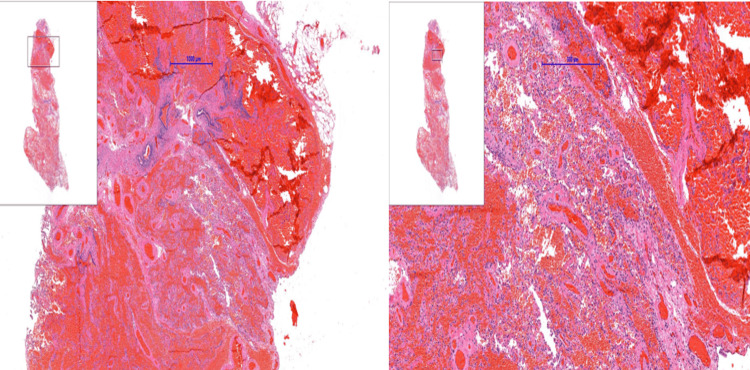
Extensive and scattered areas of ischemic infarction At the periphery of the pulmonary parenchyma in the surgical specimen, a well-defined lesion is observed, measuring 3.5 mm at its greatest dimension under microscopy. The lesion is characterized by pneumocytic proliferation with mild to moderate atypia, increased cell size, elevated nuclear-to-cytoplasmic ratio, and hyperchromatic nuclei.

In addition, the specimen showed scattered ischemic infarcts with incomplete organization (Figure 4). Given the discrepancy between the initial biopsy and the surgical pathology findings, an external review of the resected specimen slides was requested. The review confirmed the diagnosis of AAH.

## Discussion

Epidemiological data reveal compelling patterns in the occurrence of AAH, particularly in relation to lung cancer. Comprehensive surgical series have reported the presence of AAH in 5%-20% of resected lungs with pulmonary carcinoma, with a notable sex disparity: AAH was found more frequently in women (30.2%) than in men (18.8%) with ADC [1-5]. AAH also shows a strong association with primary lung carcinomas, appearing in 20% of such cases, compared to only 4.8% in non-primary tumors [3], underscoring its possible role in primary lung cancer development.

This association is clinically significant, as studies estimate a 10%-15% chance of malignancy coexisting with or arising from AAH [1]. AAH is now recognized not only as a precursor to ADC but also as a lesion that may coexist with it [6]. Travis et al. illustrated this point by documenting 16 patients with ADC who also had concomitant AAH [7]. These findings have helped establish criteria for classifying precursor lesions, which include the following: the lesion must precede malignancy development, show consistent cellular atypia or molecular changes, and occur more frequently than the associated carcinoma [7].

Treatment strategies for AAH vary widely, reflecting the complexity of its management. Some cases can be managed conservatively with close imaging surveillance, while others may require surgical intervention, such as wedge resection or lobectomy [8]. The potential role of stereotactic body radiation therapy remains under investigation, though it may offer a non-surgical option for patients who are either medically inoperable or decline surgery [1].

In this case, the detection of a KRAS G12A mutation further complicates the clinical picture. KRAS mutations are common in non-small cell lung cancer and are typically associated with poorer prognosis and fewer targeted treatment options. Unlike the more well-studied G12C variant, G12A is currently untargetable, and its presence, alongside low PD-L1 expression, suggests limited benefit from immunotherapy. As demonstrated by Sun et al., non-G12C KRAS mutations correlate with shorter progression-free survival, reinforcing the appropriateness of definitive surgical management for this patient [9].

As previously mentioned, the discrepancy in the pathological findings prompted a review of the surgical specimen slides to better clarify the true nature of the patient's tumor, given the significant implications for treatment planning and follow-up care. A similar situation was reported by Balasubramanian et al., where AAH was initially misdiagnosed as ADC [10].

In our case, the management approach might have differed if the initial histological diagnosis had been AAH rather than ADC, potentially favoring a more conservative strategy with close imaging surveillance. However, it is also reasonable to manage AAH similarly to early-stage ADC, which may have still led to surgical resection and, ultimately, the same clinical outcome.

Nevertheless, follow-up protocols differ substantially between these two entities. For ADC, surveillance typically involves chest CT scans every three months for the first two years, followed by scans every six months up to five years post-treatment. In contrast, patients diagnosed with AAH may only require annual CT scans [11].

Ultimately, this case highlights an underreported but clinically significant scenario: diagnostic discordance between biopsy and surgical specimen findings. While the initial biopsy showed features consistent with ADC, histological evaluation of the resected specimen revealed only AAH. This unexpected discrepancy prompted a thorough pathological review, which attributed the findings to dispersed ischemic infarcts with incomplete organization, potentially representing regressed areas of ADC no longer detectable, leaving only AAH tissue behind. Although direct evidence of infarction masking ADC is limited, Fraire and Greenberg suggested that ischemic changes, alveolar collapse, or organizing infarcts within tumors may contribute to the focal regression of malignant components. In such cases, histological evaluation may reveal only AAH-like or fibrotic alterations without overt evidence of adenocarcinoma, particularly when the lesion has undergone partial necrosis or spontaneous regression [12].

## Conclusions

This case underscores the diagnostic challenges of small pulmonary nodules with radiological features suggestive of malignancy. The discordance between the biopsy result (adenocarcinoma) and the surgical specimen (AAH) highlights the limitations of percutaneous sampling and the potential impact of lesion heterogeneity or regression. It emphasizes the importance of a multidisciplinary approach, second-opinion pathology when indicated, and careful correlation of imaging and histological findings. Ultimately, an accurate diagnosis is essential, not only to guide appropriate treatment but also to determine the intensity of follow-up, helping to avoid both overtreatment and delayed intervention for malignancy.
